# Energy Adequacy of Planned Diets in Institutionalized Older Adults: A Scenario Analysis Based on Requirements from Indirect Calorimetry

**DOI:** 10.3390/nu18050783

**Published:** 2026-02-27

**Authors:** Michał Ławiński, Natalia Grodzicka, Agnieszka Pawłowska-Muc, Kinga Biernacka-Stefańska, Krzysztof Ławiński, Mirosław Perliński, Katarzyna Zadka

**Affiliations:** 1 Department of General, Gastroenterology, and Oncologic Surgery, Medical University of Warsaw, Banacha 1a, 02-097 Warsaw, Poland; michal.lawinski@wum.edu.pl; 2 Department of Biotechnology and Nutrigenomics, Institute of Genetics and Animal Biotechnology, Polish Academy of Sciences, Postępu 36a, Jastrzębiec, 05-552 Magdalenka, Poland; 3 Independent Researcher, 01-931 Warsaw, Poland; 4 Department of Nursing, Faculty of Health Sciences, The Józef Goluchowski University of Applied Sciences, Akademicka 12, 27-400 Ostrowiec Świętokrzyski, Poland; 5 Independent Public Healthcare Complex of Lech and Maria Kaczyński, Niepodległości 1, 26-670 Pionki, Poland; 6 Department of Dietetics, Institute of Human Nutrition Sciences, Warsaw University of Life Sciences/SGGW, Nowoursynowska 159c, 02-776 Warsaw, Poland; 7 University Clinical Centre, Dębinki 7, 80-952 Gdańsk, Poland; 8 Faculty of Pharmacy, University of Vizja, Okopowa 59, 01-043 Warsaw, Poland

**Keywords:** older adults, malnutrition, healthy aging, nutritional screening, nutrition planning

## Abstract

**Background/Objectives:** Older adults living in long-term care facilities (LTCFs) are at high risk of undernutrition. This study evaluated the adequacy of planned energy intake (PEI) by comparing prescribed diets with individual requirements measured using indirect calorimetry (IC) and by modelling how different levels of food consumption affect energy adequacy. **Methods:** In this cross-sectional study, 169 adults aged ≥ 65 years living in LTCFs underwent anthropometric assessment and IC-based measurement of resting energy expenditure. Total energy expenditure (TEE) was derived using activity-specific PAL factors. PEI was calculated from 7-day menu records (oral diets) or enteral feeding prescriptions. Scenario analyses assumed intake levels from 100% to 50% of PEI and applied BMI-specific adequacy thresholds. **Results:** Mean TEE was 1447 ± 359 kcal/day (25 ± 6 kcal/kg), whereas mean PEI was 1999 ± 400 kcal/day, yielding an average surplus of 552 ± 496 kcal/day and a TEE/PEI ratio of 0.76. PEI did not differ across sex, BMI, or activity groups despite significant differences in measured TEE. Individuals receiving enteral nutrition demonstrated close agreement between intake and expenditure. Fewer than half of residents consumed > 75% of their served portion, about one third consumed 51–75%, and approximately one fifth consumed ≤ 50%, based on caregiver reports. Scenario modelling showed that the proportion of adults meeting adequacy criteria remained relatively stable at intake levels of 100–70% of PEI but declined significantly below 70%. **Conclusions:** Planned dietary energy provision exceeded measured requirements, yet underweight remained frequent, indicating a gap between prescribed and consumed energy. Monitoring actual intake and adjusting provision to individual needs are essential in LTCFs.

## 1. Introduction

Malnutrition is recognized as an issue in the increasing global aging population [1]. The International Association of Gerontology and Geriatrics, together with the International Academy of Nutrition and Aging, identify multiple risk factors for malnutrition in older adults. Key factors include very advanced age (≥85 years) and inadequate nutrient intake resulting from loss of independence during eating, as well as swallowing and chewing impairments. In addition, older adults with frailty often present with multiple clinical, functional, and psychological conditions. These commonly include cognitive decline, depressive symptoms, chronic comorbidities, and complications associated with immobility [2]. The prevalence of malnutrition and risk of malnutrition in older adults varies considerably across settings, affecting approximately 30–60% of hospitalized patients and 47–85% of those in long-term care institutions [3,4,5].

Inadequate dietary intake and the resulting malnutrition are widely observed among older individuals residing in long-term care facilities (LTCFs) [4]. Older adults living LTCFs represent a clinically distinct population compared with older adults residing in the community. Facility residents typically require extensive assistance with daily activities and frequently exhibit multifaceted health conditions, such as cognitive impairment, neurological diseases, diabetes, and cardiovascular disorders [6]. These coexisting conditions not only define their medical status and nutritional demands but are also associated with significant functional decline, limited mobility, and substantially reduced physical activity [7]. Consequently, adults living in LTCFs commonly demonstrate more rapid deterioration of skeletal muscle mass affecting energy needs, a greater burden of frailty, and a markedly elevated susceptibility to malnutrition relative to their community-dwelling peers, as consistently reported in the recent literature [2,8,9]. These alterations occur alongside a progressive decline in muscle function; together, the loss of muscle mass and function corresponds to the clinical features of sarcopenia frequently observed in this population. Importantly, sarcopenia itself is strongly linked with nutritional risk, indicating a bidirectional relationship in which impaired nutritional status and adverse body composition mutually reinforce functional decline [10]. In addition body weight loss and impaired nutritional status are associated with a higher incidence of falls, an increased prevalence of chronic wounds, delayed wound healing, more frequent hospitalizations, and reduced quality of life [11]. It should be added that unintentional weight loss in older adults should be regarded as clinically concerning even when BMI remains above established diagnostic thresholds, including in individuals with obesity [6].

Importantly, malnutrition in older adults extends beyond deficits in body weight or energy balance and frequently includes micronutrient inadequacy. Micronutrient deficiencies represent a highly prevalent yet often under-recognized component of malnutrition in this population, as clinical attention tends to focus primarily on weight loss or insufficient caloric intake [12]. Recent studies in institutionalized and hospitalized older adults have demonstrated that multiple micronutrient deficiencies frequently coexist, even in individuals receiving standard institutional diets or supplementation [12,13,14]. Limited dietary diversity, impaired eating ability, multimorbidity, and polypharmacy further increase the risk of inadequate intake and altered nutrient absorption, contributing to subclinical deficiencies that may adversely affect functional status and overall health [13,15]. Current clinical guidelines emphasize that older adults, particularly those with chronic diseases and frailty, are at increased risk of deficiencies in vitamin D, vitamin B12, folate, iron, calcium and other micronutrients [15], and recommend systematic assessment in individuals at nutritional risk [16].

Apart from individual clinical factors, the long-term care setting itself may additionally shape energy requirements and dietary adequacy [17]. In LTCFs, nutritional care is frequently marked by variability in energy and nutrient consumption, reliance on feeding assistance, and complications such as dysphagia, all of which may exacerbate metabolic dysregulation [18,19]. Residents of LTCFs are also more commonly affected by polypharmacy, repeated minor infections, and persistent low-grade inflammation, factors that can alter metabolic needs in both increasing and decreasing directions. This variability complicates nutritional planning and may consequently elevate the risk of malnutrition [20,21,22].

In this context, the concept of precision and personalized nutrition becomes particularly relevant. Adequate energy intake is particularly important in this population, as insufficient energy supply results in the use of protein for energy production rather than for structural and functional purposes, thereby contributing to the development of sarcopenia [23]. Estimation of energy requirements in older adults is commonly based on simplified weight-based rules of thumb (30 kcal/kg body weight) [15] or predictive equations; however, their accuracy in frail, multimorbid, and functionally dependent populations remains limited [24,25]. Age-related changes in body composition, inflammatory status, comorbidities, and variability in resting energy expenditure (REE) may lead to substantial over- or underestimation of true energy needs when indirect methods are applied [17]. Indirect calorimetry (IC) therefore represents the reference method for assessing individual energy expenditure [26,27] and may provide a more reliable basis for evaluating dietary adequacy in institutionalized older adults.

The aim of this study was to evaluate the adequacy of planned diets for institutionalized older adults by comparing prescribed energy intake (PEI) with individual requirements derived from IC. In addition, scenario-based analyses (100–50% of PEI) were applied to examine how varying levels of food consumption would affect the proportion of residents achieving clinically appropriate energy intake, taking into account nutritional status.

## 2. Materials and Methods

### 2.1. Study Design and Ethics Approval

This cross-sectional study was performed in four LTCFs located in the Masovian region of Poland. One facility was situated in a metropolitan city with a population exceeding one million, whereas the other three were in smaller towns ranging from 15,000 to 70,000 residents, all positioned within a 130 km radius of the large urban center. To ensure methodological consistency, all measurements were performed by the same trained research team using standardized procedures. Due to financial and logistical constraints associated with repeated assessments, the study was conducted in geographically accessible LTCFs that agreed to participate and met the technical requirements for implementation of the protocol. The selection of facilities was therefore based on feasibility and institutional willingness to collaborate.

The study was conducted in line with the ethical principles outlined in the Declaration of Helsinki and received approval from the Bioethics Committee at the Medical University of Warsaw (approval no. KB/37/2025). Participant enrollment was carried out over a two-month period, from April to June 2025.

### 2.2. Study Population

The study population consisted of older adults aged 65 years and above residing in LTCFs. Inclusion criteria were as follows: age over 65 years, a minimum of three months of residence in an LTCF, clinically stable health status, absence of acute infections or exacerbations of chronic diseases within the preceding two weeks, no active oncological treatment or a history of completed cancer therapy at least five years before enrollment, receipt of oral, enteral, or mixed nutrition, and preserved consciousness or the possibility of obtaining informed consent from a legal guardian. Exclusion criteria included terminal illness, claustrophobia, dependence on parenteral nutrition, or unconsciousness with no option for surrogate consent. Prior to participation, all residents or their caregivers received detailed information about the study protocol and were asked to provide written informed consent. Participation was voluntary, and withdrawal from the study was permitted at any time without the need to state a reason.

**Figure 1:**
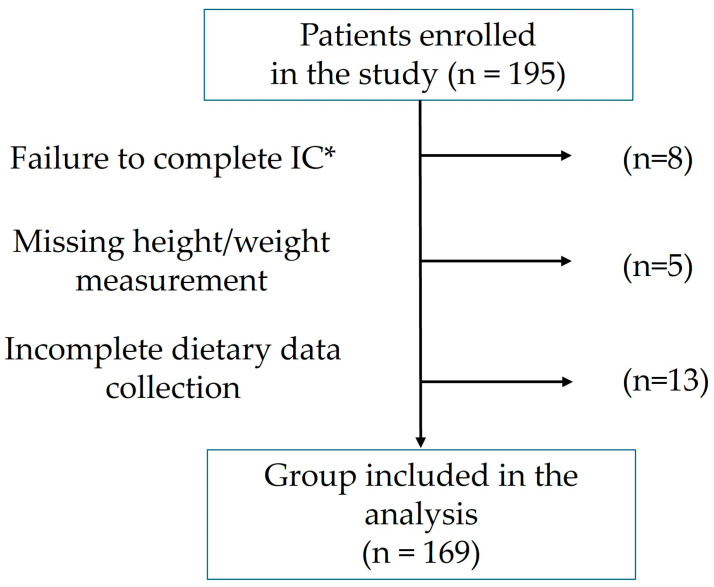
Flow chart showing participant dropout. * IC—indirect calorimetry.

A total of 195 older adult residents of LTCFs (71.3% women) were enrolled in the study, with a mean age of 81.7 ± 9.2 years. Twenty-six participants did not complete the study for the following reasons: 8 did not undergo IC, 5 could not be weighed or measured due to medical contraindications, and in 13 cases, records of weekly dietary intake or enteral feeding were incomplete, which precluded assessment of dietary energy content (Figure 1).

### 2.3. Measurements

All assessments were carried out within the respective LTCFs of residence using standardized equipment and harmonized measurement procedures across sites.

#### 2.3.1. Anthropometry

Body weight assessment was carried out using different approaches depending on the resident’s condition. Those who were able to stand unaided were weighed with a leveled, calibrated electronic scale (WB-380, TANITA, Tokyo, Japan; accuracy 0.1 kg). For participants unable to stand, a lift scale was applied. Each measurement was documented immediately after completion.

Stature was measured with a precision of 0.5 cm. Standing height, whenever feasible, was taken with the stadiometer integrated into the scale. In adults for whom this was not possible, supine length was obtained with a sliding scale. If neither method could be applied, the most recent data from medical records were used.

Nutritional status was classified using Body Mass Index (BMI) with age-adapted cut-offs applied for older adults: underweight (<20 kg/m^2^ for participants < 70 years; <22 kg/m^2^ for participants ≥ 70 years), normal weight (20–25 kg/m^2^ or 22–27 kg/m^2^), overweight (25–30 kg/m^2^ or 27–30 kg/m^2^), and obesity (≥30 kg/m^2^), consistent with previously published approaches in geriatric populations [28,29,30].

Mid-upper arm circumference (MUAC) was assessed using a non-stretchable measuring tape and a standardized protocol. With participants seated or lying comfortably and both arms relaxed, the midpoint between the acromion and the olecranon on the non-dominant arm was identified. At this level, the arm circumference was measured without applying pressure to the subcutaneous tissue. Each measurement was repeated three times, and the mean value was used for analysis. In accordance with previous research, cut-off values indicating risk of undernutrition in older adults were defined as <23.5 cm for men and <22.0 cm for women [31].

#### 2.3.2. Assessment of Resting and Total Energy Expenditure

REE was evaluated in all participants by IC with the Q-NRG device (COSMED, Rome, Italy). Following the manufacturer’s instructions, the system was switched on in advance and calibrated approximately 20 min after activation, prior to initiating measurements. The procedure was carried out in line with the ICALIC study group recommendations [27], requiring at least 5 h of fasting, avoidance of alcohol and nicotine, measurement in a supine but awake state, and the provision of a thermoneutral and stress-free environment. Before data acquisition, participants remained quietly in bed for 15 min to allow for adaptation. Measurements were performed with a canopy hood system equipped with a disposable filter and protective covering. After a short automatic calibration (~2 min), data were collected for 30 min. Results were expressed to the nearest kilocalorie, and REE was derived using the abbreviated Weir equation [32].

Total energy expenditure (TEE) was estimated by multiplying the measured REE by an appropriate physical activity level (PAL) factor. Classification was based on direct observation of residents’ daily functioning and structured consultation with nursing staff, and PAL values were selected according to literature-based ranges corresponding to the mobility profile of each individual LTCF resident. A PAL of 1.20 was applied for participants confined to bed or sitting out, 1.25 for those with limited mobility within the LTCF, and 1.30 for residents with a sedentary lifestyle in the LTCF [33]. Activity level was classified according to functional characteristics. The bed or seated category included residents who spent most of the day confined to bed or seated and did not ambulate independently within the facility. Limited mobility referred to residents who were able to stand and walk short distances but required staff assistance or used mobility aids such as a walker or wheelchair, typically presenting with slow gait speed, reduced endurance, and dependence in basic daily tasks. The sedentary category comprised individuals who could walk short distances independently within the facility but exhibited minimal spontaneous physical activity. TEE was expressed as total daily energy expenditure and relative to body weight (kcal/kg).

### 2.4. Assessment of Planned Energy Intake (PEI) in Oral and Enteral Nutrition

For adults living in LTCFs receiving oral nutrition, planned energy intake (PEI) was calculated from 7-day menu records corresponding to the diets individually planned and provided to each resident in the LTCF, as documented by the facility dietitian. The caloric content of each day’s menu, including all meals and beverages, was calculated separately, and the mean across the 7 days was used as the individual’s average daily PEI. PEI was also expressed relative to body weight (kcal/kg). In addition, the absolute difference (Δ = PEI − TEE) was calculated, and the ratio of TEE to PEI was used to indicate the proportion of the planned menu that would need to be consumed to fully meet individual TEE. Caregivers from LTFCs reported the approximate proportion of meals usually eaten by each resident; however, these reports referred only to meals as a whole and served as a very general indication of intake, since they were not based on precise weighing or detailed assessment of individual food components. Because of these limitations, the information could not be used directly to quantify actual consumption.

As a result, analytical scenarios were applied to account for potential variability in actual intake. Scenarios were constructed assuming consumption of 100%, 90%, 80%, 70%, 60%, and 50% of PEI. For each scenario, adequacy was evaluated by dividing the assumed energy intake by TEE, with classification dependent on BMI category. Residents classified as overweight or obese according to BMI were excluded from this analysis, as in some cases the prescribed diet intentionally involved a caloric deficit. For residents with underweight, intake was considered adequate when the intake-to-TEE ratio exceeded 0.9. Among residents with normal body weight, adequacy was defined as a ratio between 0.9 and 1.1, while values above 1.1 were classified as excessive. Regardless of BMI category, values below 0.9 were considered insufficient, consistent with prior definitions of energy intake adequacy in institutionalized older adults [34].

In adults receiving enteral nutrition, energy intake was calculated as the product of the caloric density of the prescribed industrial formula and the administered volume across seven consecutive days. Since all participants in this group received the full prescribed regimen (100%), they were not included in the scenario-based analyses.

### 2.5. Statistical Analysis

Descriptive statistics were applied to characterize the study population. Categorical variables were expressed as counts and percentages. All distributed variables were presented as mean (M) with standard deviation (SD). The distribution of continuous variables was examined using the Shapiro–Wilk test. Comparisons between two groups were carried out using the *t*-test or the Mann–Whitney U test, depending on the distribution of the data. For comparisons across more than two groups, one-way ANOVA was used when normality was confirmed. The homogeneity of variances was verified using Levene’s test, and in cases where this assumption was not met, Welch’s ANOVA was applied. When the normality assumption was violated, the Kruskal–Wallis test was used instead. Post hoc pairwise comparisons were conducted using Tukey’s HSD test after one-way ANOVA, Bonferroni-adjusted *t*-tests following Welch’s ANOVA, and Dunn’s test with Bonferroni correction after the Kruskal–Wallis test. Associations between categorical variables were examined using the Chi-square (χ^2^) test of independence.

To evaluate whether the proportion of residents classified as having clinically appropriate intake differed across hypothetical consumption scenarios (100% to 50% of PEI), adequacy was recoded into binary variables (appropriate = 1, not appropriate = 0). Adequacy was defined in a BMI-specific manner: for underweight residents, intake ≥ 0.9 TEE was considered appropriate, while for residents with normal body weight, adequacy was defined as intake within 0.9–1.1 TEE. A global comparison across all six scenarios was performed using Cochran’s Q test for repeated binary outcomes. Pairwise differences between adjacent scenarios were subsequently examined with McNemar’s test for paired proportions. Results are reported as χ^2^ statistics with corresponding *p*-values. For multiple pairwise comparisons, unadjusted *p*-values are presented, with interpretation focused on consistent patterns of change.

Statistical analyses were performed using STATISTICA software, version 13 (TIBCO Software Inc., Palo Alto, CA, USA), and a *p*-value of <0.05 was considered statistically significant.

## 3. Results

### 3.1. Participant Characteristics

The study included 169 adults living in LTCFs, of whom 73.4% were women. The mean age of the study group was 82 ± 9 years. Women were significantly older than men (*p* < 0.001), most frequently aged 85–94 years, whereas men were most often in the 65–74 year group (Table 1).

Two thirds of participants were bedridden or sitting out, and oral feeding predominated. Men were also significantly taller and heavier than women (*p* < 0.001). According to the age-adopted BMI cut-offs applied in this study, nearly half of the residents were underweight, with this condition more common among women, while overweight and obesity were relatively more frequent among men. BMI did not differ significantly between sexes. MUAC-defined undernutrition was present in one quarter of the study group and occurred more often in women. Based on caregiver-reported usual intake, fewer than half of residents were judged to consume > 75% of the served portion, about one third 51–75%, and roughly one fifth ≤ 50%. Men were more likely than women to fall in the >75% category, whereas lower consumption categories were more common among women. Detailed characteristics of the study population are presented in Table 1.

### 3.2. Resting and Total Energy Expenditure, Prescribed Intake, and Adequacy Ratios

In the total study population, PEI exceeded TEE by a mean of 552 ± 496 kcal/d, with an average TEE/PEI ratio of 0.76 ± 0.24. This indicates that, in the absence of a need for energy supply modification, consumption of approximately 76% of the prescribed intake would be sufficient to meet estimated requirements. TEE expressed per kilogram of body weight was 25 ± 6 kcal (Table 2).

Men had significantly higher REE and TEE than women (*p* < 0.001), but not when expressed per body weight. Since PEI did not differ between sexes, the energy surplus in men was smaller and the TEE/PEI ratio higher (*p* < 0.001).

Across BMI categories, no significant differences were observed in the absolute PEI (*p* = 0.070). However, due to differences in body weight between groups, PEI expressed per kilogram of body weight varied significantly (*p* < 0.001). PEI expressed per kilogram of body weight was highest in the underweight group, intermediate in residents with normal body weight, and lowest in those with overweight, whereas values observed in the obesity group did not differ significantly from the two intermediate categories. REE and TEE were significantly higher in overweight residents (both *p* < 0.001) compared with those who were underweight or of normal weight, while values in the obesity group did not differ significantly from any other BMI category. In contrast, TEE expressed per kilogram of body weight was significantly higher in the underweight (*p* < 0.001) group than in all other BMI categories, which did not differ from one another. The smallest difference between PEI and TEE, as well as the TEE/PEI ratio closest to 1, was observed in overweight patients (both *p* < 0.001), who differed significantly from both the underweight and normal-weight groups. Conversely, the largest differences were observed in underweight patients, both for PEI–TEE and for TEE/PEI, the latter being the most distant from 1 (both *p* < 0.001). However these values did not differ significantly from those with normal body weight.

Stratification by MUAC showed a similar trend: patients with undernutrition had lower expenditure and higher surpluses than those with normal MUAC, resulting in significantly lower TEE/PEI ratios (*p* = 0.048).

Activity level was also associated with energy balance: adults confined to bed or a chair had significantly lower REE and TEE and exhibited larger energy surpluses than both groups with higher activity, whereas those with limited mobility and sedentary residents showed comparably higher expenditure and TEE/PEI ratios approaching unity.

Feeding route had a marked effect. TEE was higher in adults receiving oral nutrition (*p* = 0.005), but TEE per kilogram of body weight did not differ between groups (*p* = 0.207). In contrast to other stratifications, prescribed intake differed substantially: PEI was nearly twice as high in adults receiving oral nutrition as adults receiving enteral nutrition. Consequently, it demonstrated a clear energy surplus, while in enterally fed patients intake and expenditure were nearly equivalent, with a mean TEE/PEI ratio of 1.0 (*p* < 0.001).

Finally, consumption of served meals was directly associated with energy balance: patients consuming ≤75% of meals had larger surpluses and lower TEE/PEI ratios, whereas those consuming >75% showed smaller surpluses and significantly higher ratios. It should be noted that among the 77 patients in the highest consumption group, 26 were receiving enteral nutrition and thus consumed 100% of their prescribed intake.

### 3.3. Scenario-Based Analysis of Intake Adequacy

For the scenario analysis, 106 LTCF residents were included (78.3% female), with a median age of 83.5 ± 8.4 years. A total of 62.3% were underweight, while the remaining participants had a normal body weight. Mean values of REE, TEE, and PEI in this subgroup (1137 ± 237, 1390 ± 306, and 2153 ± 253 kcal, respectively) did not differ significantly from those observed in the total study population. However, the difference between PEI and TEE was greater (762 ± 410 kcal/day), and the TEE-to-PEI ratio was lower (0.66) than in the overall study group.

The distribution of residents across intake adequacy categories under hypothetical food consumption scenarios, stratified by nutritional status (underweight vs. normal weight), is presented in Table 3.

In the underweight group, the proportion of residents with clinically appropriate intake was highest at 100% PEI (98.5%) and gradually declined with lower scenarios, reaching only 30.3% at 50% PEI. In the normal-weight group, the proportion classified as adequate peaked at 60–80% PEI (25–27.5%), whereas both higher and lower scenarios showed lower adequacy rates (10% at 100% PEI and 20% at 50% PEI). The prevalence of insufficient intake (<0.9 TEE) increased consistently with lower PEI in both groups, reaching 69.7% in the underweight group and 77.5% in the normal-weight group at 50% PEI.

The global test confirmed these differences: Cochran’s Q(5) = 86.3, *p* < 0.000001, indicating that changes in the proportion of residents with adequate intake across scenarios were statistically significant and not due to chance.

Pairwise McNemar tests confirmed that adequacy changed significantly only at lower intake levels. No significant differences were observed in 100% vs. 90%, 90% vs. 80%, or 80% vs. 70% PEI (all *p* > 0.05). However, a marked decline in adequacy was observed from 70% to 60% PEI (χ^2^ = 5.28, *p* = 0.022) and from 60% to 50% PEI (χ^2^ = 14.05, *p* < 0.001).

The findings demonstrate a substantial mismatch between planned energy intake and individual energy requirements in LTCF residents, driven largely by the high prescribed energy provision and the wide variability in TEE across subgroups. On average, PEI exceeded TEE by more than 550 kcal/day, yielding a TEE/PEI ratio of 0.76 and indicating that consuming approximately 76% of the planned diet would be sufficient to meet estimated needs. This imbalance was most pronounced among residents classified as underweight according to BMI and among those with the lowest activity levels, who exhibited the greatest energy surpluses. In contrast, enterally fed residents showed near alignment between PEI and TEE, with a mean ratio of 1.0, reflecting more precise tailoring of energy provision. Scenario-based modelling further revealed that intake adequacy remained stable when consumption reached at least 70% of PEI but declined sharply at lower intake levels, particularly among underweight individuals.

## 4. Discussion

This study examined older adults living in LTFCs—individuals of advanced age and with markedly limited mobility—among whom indicators of undernutrition were common and meal consumption was frequently incomplete. Residence in LTCFs becomes more common with advancing age, especially during the final years of life [35]. The mean age of residents in our LTCF cohort was similar to that reported in Norwegian cohorts (84 y) [36], and Dutch LTCF studies (85 y) [6]. In the examined population, most residents were women, and the age distribution differed markedly by sex. This pattern is consistent with women’s longer life expectancy and their higher likelihood of entering long-term care [37,38]. In Poland in 2024, average life expectancy was 74.93 years for men and 82.26 years for women, according to the Central Statistical Office of Poland [39].

Malnutrition in older adults is a major geriatric concern, given its high prevalence, multifactorial etiology, and serious health consequences [3]. Nearly half of our residents were underweight (46.2%), exceeding rates reported in Norwegian, Swedish, Dutch, and German cohorts (33%, 30%, 27.4%, and 13.7%, respectively) [6,36,40,41]. The lower prevalence observed in the German study was partly due to the use of different BMI criteria. However, even when applying the same criteria to our population, the prevalence of underweight remained higher than in the German cohort.

In LTCFs, accurate assessment of energy requirements is particularly important, as standardized predictive approaches may fail to capture the metabolic heterogeneity observed among institutionalized and hospitalized older adults. Studies applying IC in these populations have consistently demonstrated substantial interindividual variability in REE and have shown that commonly used predictive equations may perform poorly in these settings, frequently underestimating or overestimating true energy expenditure [1,17,20]. Despite its status as the reference method for assessing REE and its alignment with precision nutrition principles, routine implementation of IC in long-term care settings may be constrained by equipment investment, operational costs, and the need for trained personnel [42,43]. In this context, prioritizing its use in clinically complex or high-risk residents may represent a more feasible strategy.

In our study IC revealed that among examined older adults living in LTFCs, TEE was relatively low, averaging about 1450 kcal/day, or approximately 25 kcal/kg, reflecting minimal physical activity levels. Our findings are consistent with conclusions from a systematic review indicating that mean daily energy intake among LTCF residents often ranges between 1250 and 1800 kcal/day [44]. The energy expenditure observed in our study population aligns well with current European Society for Clinical Nutrition and Metabolism concepts, which indicate that REE in older individuals typically approximates 20 kcal/kg/day and, when combined with physical activity levels, yields TEE ranging from roughly 24 to 36 kcal/kg/day [15]. The mean TEE of approximately 25 kcal/kg/day identified in our residents is therefore consistent with the lower end of this spectrum, reflecting the very limited mobility characteristic of many individuals living in long-term care. The planned diets among our residents provided roughly 2000 kcal/day (about 36 kcal/kg), resulting in an average surplus exceeding 550 kcal/day. Gaillard et al. further notes that REE expressed per kilogram increases as BMI decreases, and that older adults with underweight may require 32–38 kcal/kg/day to prevent further nutritional decline [45]. In this context, the planned energy provision of about 36 kcal/kg/day appears reasonable in a population with a high prevalence of underweight, as it corresponds to the upper range of requirements recommended for individuals with low BMI.

The marked divergence between PEI and TEE in our study indicates that uniform energy prescriptions do not adequately account for the heterogeneity of functional status and food intake. This interpretation is further supported by the absence of statistically significant differences in prescribed energy intake between groups, including comparisons by sex, BMI category, and activity level. Taken together, these findings suggest that energy provision in the participating LTCFs was largely menu-driven rather than needs-driven, with prescribed diets reflecting standardized meal planning at the institutional level rather than systematic adjustment to individual metabolic requirements and functional status, despite clear differences in measured energy expenditure between these same subgroups.

In our study among residents receiving oral nutrition a mean TEE/PEI ratio of 0.71, suggesting that consuming around 70% of the prescribed diet would theoretically cover individual energy needs. Scenario modelling further indicated that energy adequacy was generally maintained at intake levels of at least 70% of PEI, so among our residents planned diet should cover needs. The modelling approach assumed proportional reductions in planned intake and did not account for potential nonlinear or dynamic patterns of real-life food consumption; therefore, the 70% level should be interpreted as an inferential estimate rather than a definitive clinical threshold. However caregiver reports suggested that many residents routinely consumed ≤75% of their meals, placing a substantial proportion of residents close to a threshold at which even modest additional reductions in intake may result in inadequate energy coverage. Also in a new systematic review in more than 30% of analyzed studies actual intake was <75% of the energy planned in the menu, reflecting widespread inadequacies in energy and nutrient provision in long-term care settings [44].

Habitual consumption of less than 75% of meals among LTCF residents may explain why, despite PEI exceeding TEE in our study population, the prevalence of underweight remained high. This apparent paradox has been described in previous research, and suggests that providing energy alone may not suffice to guarantee adequate nutritional status. For example, plate-waste studies among older adults living in residential care have documented substantial losses between served meals and actual consumption, indicating that the energy “offered” often does not equal the energy “ingested” [46]. Findings from different long-term care settings illustrate how consistently this pattern appears across countries and facilities. Earlier work from a 140-resident facility in the United States showed that roughly one-fifth of all food provided was not consumed [47]. More recent Australian investigations reported that residents left on average 91 g of each main meal uneaten, corresponding to approximately 273 g of food wasted per person per day [48]. Comparable observations were made in Canada, where nearly one-third of food and beverage items distributed in an Ontario residential facility were discarded, with lunch showing the highest losses. The extent of waste also varied markedly between residents, with about 15% discarding more than half of the items offered [49]. Belgian studies add further nuance: precise weighing demonstrated that residents consumed only about 1550 kcal of nearly 1800 kcal provided daily [50], and institutional audits estimated total food losses between 25% and 40%, equating to roughly 287 g of food wasted per resident per day [51]. Additional evidence confirms the magnitude of this issue: mean plate waste amounted to 112.3 ± 35.2 g per resident per day, equivalent to approximately 10.9% of total food served. Items most commonly left uneaten included bread, meat or fish, and starchy components. Importantly, preparation losses at lunch alone reached 37.8%, corresponding to nearly 193 g per resident per day—showing that a substantial portion of nutrients never reaches the plate in the first place [52]. A complementary perspective is provided by Nanayakkara et al. [46], who demonstrated that nutrient losses accumulate at multiple stages—from planned menu, to prepared meal, to served portion, to what is ultimately consumed—highlighting that menu-based energy estimates frequently overstate actual intake in aged-care residents.

A notable observation from our study is that residents receiving enteral nutrition had a closer alignment between prescribed energy and measured expenditure, compared to those on oral diets. This suggests that enteral nutrition protocols—often more standardized and tailored to individual requirements—may more reliably meet energy needs in populations with low intake or impaired intake capacity. However, residents receiving tube feeding often represent a clinically more complex subgroup, and therefore closer control of delivery should be interpreted primarily as improved predictability of provision rather than as assurance of restoring a healthy body weight [18]. The available evidence does not consistently demonstrate clear benefits of enteral feeding—such as improvements in biochemical parameters, body weight, or broader clinical outcomes—particularly among older adults with multiple comorbidities or advanced disease [53]. For residents managed with oral diets, interventions that increase the likelihood that planned nutrients are actually consumed remain essential [54]. In addition to systematic monitoring of intake and staff-supported feeding, oral nutritional supplements may serve as a practical dietary adjunct when meals alone are not reliably consumed [55], because they can increase total energy and protein intake without requiring large meal volumes [56], although high-quality outcome trials specifically in long-term care settings remain comparatively limited [57].

In light of the observed mismatch between planned energy provision and both measured requirements and actual intake, routine nutritional care in LTCFs should extend beyond menu-based planning and incorporate systematic, individualized monitoring. This includes regular screening for undernutrition risk, periodic reassessment of energy and protein needs—preferably using objective methods or validated predictive tools—and meticulous monitoring of actual food intake rather than reliance on planned menus alone. Meal plans should be dynamically adjusted according to measured requirements, functional status, and observed intake patterns. For residents with persistently insufficient oral intake, early consideration of oral nutritional supplementation or enteral nutrition within a structured care protocol is warranted. An interdisciplinary approach involving dietitians, nursing staff, and physicians is essential to ensure that nutrition therapy is continuously adapted to residents’ changing clinical and functional status.

A major strength of this study is the use of IC, the reference method for assessing REE in clinical settings, enabling precise estimation of individual energy requirements among institutionalized older adults. Combined with comprehensive assessment of anthropometric indicators, activity level, and feeding route, this approach allowed for a detailed characterization of heterogeneity in energy needs across subgroups. The application of scenario-based modelling provided additional insight into how varying levels of meal consumption may influence the adequacy of energy intake—an issue highly relevant to long-term care practice. Conducting assessments within residents’ habitual environment and using consistent staff and standardized procedures further strengthened internal validity and reduced measurement variability.

Several limitations should be considered. First, actual dietary intake could not be quantified objectively, as caregiver-reported estimates of portion consumption lacked direct weighing. Moreover, caregiver-reported intake was based on visual estimation of meal completion rather than standardized weighing procedures, which may introduce inter-observer variability and limit precision of intake assessment. Consequently, intake could not be incorporated as measured data, and the analyses relied on hypothetical intake scenarios that may not fully reflect real-world eating patterns. Second, the estimation of TEE relied on predefined PAL categories rather than objective individual measurements of physical activity. Although PAL classification was based on functional assessment and consultation with nursing staff who routinely observe residents, it relied on interview-based and observational data rather than direct monitoring tools such as accelerometry. Therefore, some degree of misclassification of habitual activity cannot be excluded, particularly given that predefined PAL categories may not fully capture interindividual variability in spontaneous movement and day-to-day functional fluctuations, which may have introduced measurement variability in TEE estimation. Third, the selection of LTCFs was based on feasibility and institutional agreement to participate, and all measurements were conducted within one geographic region. Although this approach ensured methodological standardization, it may limit the generalizability of the findings to LTCFs operating under different organizational, economic, or healthcare system conditions. Future multicenter studies conducted across diverse long-term care settings and healthcare systems are warranted to enhance external validity and confirm the broader applicability of these findings. Fourth, the cross-sectional design precludes conclusions about causal relationships between dietary planning, energy balance, and clinical outcomes. Fifth, nutritional adequacy in this study was evaluated primarily on the basis of energy intake relative to estimated requirements, rather than through assessment of macronutrient composition or specific nutrient adequacy. In particular, protein intake and micronutrient status were not systematically evaluated, which may limit the ability to fully characterize the multidimensional nature of malnutrition in this population. Consequently, the present findings relate predominantly to energy adequacy and may not capture qualitative aspects of dietary insufficiency that are clinically relevant in older adults residing in LTCFs.

Although IC and modelling provided valuable insight into discrepancies between planned and measured energy needs, prospective interventional studies are required to determine whether adjusting dietary plans according to measured requirements and/or documented intake leads to improvements in nutritional status, body composition, functional outcomes, and morbidity or mortality. Future research should also focus on developing practical and cost-effective tools for estimating energy needs when IC is not available, potentially integrating functional assessment, body composition measures, and objective activity monitoring.

## 5. Conclusions

In the studied long-term care residents with markedly limited mobility, measured energy expenditure was low, whereas planned dietary energy provision substantially exceeded individual requirements. Nevertheless, indicators of undernutrition were frequent, pointing to a discrepancy between energy prescribed and energy actually consumed. Prescribed energy intake did not differ across sex, BMI, or activity groups, despite significant differences in measured energy expenditure, suggesting that dietary planning did not fully reflect individual metabolic needs. Residents receiving enteral nutrition showed closer agreement between prescribed intake and measured requirements than those fed orally. Together, these findings underline the importance of monitoring actual intake and better matching dietary provision to individual energy needs in long-term care settings.

## Figures and Tables

**Table 1:** Detailed description of study population that completed the study (*n* = 169).

Variable		Total (*n* = 169)			Men (*n* = 45)			Women (*n* = 124)			*p* *
		*n*	%	M ± SD	*n*	%	M ± SD	*n*	%	M ± SD	
Age [years]	65–74	44	26.0	82.08 ± 8.73	25	55.6	75.49 ± 7.81	19	15.3	84.48 ± 7.78	<0.001
	75–84	44	26.0		13	28.9		31	25.0		
	85–94	74	43.8		7	15.6		67	54.0		
	95+	7	4.1		0	0		7	5.6		
Level of activity	In bed or sitting out	110	65.1	-	28	62.2	-	82	66.1	-	0.894
	Limited mobility	35	20.7		10	22.2		25	20.2		
	Sedentary	24	14.2		7	15.6		17	13.7		
Route of nutrition	Oral	143	84.6	-	37	82.2	-	106	85.5	-	0.603
	Enteral	26	15.4		8	17.8		18	14.5		
Body weight [kg]		-	-	59.41 ± 14.54	-	-	66.65 ± 15.80	-	-	56.78 ± 13.17	<0.001
Height [cm]		-	-	160.16 ± 10.71	-	-	171.36 ± 8.24	-	-	156.010 ± 8.36	<0.001
BMI ^1^ [kg/m^2^]	Underweight	78	46.2	23.17 ± 5.21	20	44.4	22.73 ± 5.19	58	46.8	23.33 ± 5.24	0.498
	Normal	51	30.2		11	24.4		40	32.3		
	Overweight	25	14.8		11	24.4		14	11.3		
	Obesity	15	8.9		3	6.7		12	9.6		
MUAC ^2^ [cm]	Undernutrition	43	25.4	25.52 ± 5.00	9	20.0	27.31 ± 4.60	34	27.4	24.87 ± 5.00	<0.001
	Normal	126	74.6		36	80.0		90	72.6		
Served food consumption [%]	<25	2	1.2	70.72 ± 20.25	0	0	76.22 ± 20.37	2	1.6	68.71 ± 19.92	0.042
	25–50	36	21.3		10	22.2		26	21.0		
	51–75	54	32.0		8	17.8		46	37.1		
	>75	77	45.6		27	60.0		50	40.3		

^1^ Age-adopted BMI category cut-offs: underweight (BMI < 20 kg/m^2^ if <70 years and <22 kg/m^2^ if ≥70 years), normal body weight (BMI 20 to 25 kg/m^2^ if <70 years and 22 to 27 kg/m^2^ if ≥70 years), overweight (BMI 25 to 30 kg/m^2^ if <70 years and 27 to 30 kg/m^2^ if ≥70 years), obesity (BMI > 30 kg/m^2^); ^2^ MUAC—mid-upper arm circumference, undernutrition was defined as <23.5 cm for men and <22.0 cm for women. The values do not add to 100% because of rounding. * U Mann–Whitney test or χ^2^ depending on the type and distribution of the variable.

**Table 2:** Resting (REE) and total energy expenditure (TEE), planned energy intake (PEI), PEI-TEE difference, and TEE/PEI ratio in the study population (*n* = 169) and subgroups.

Variable ^1^	REE ^2^ [kcal/d]	TEE ^3^ [kcal/d]	TEE ^3^ [kcal/kg BW]	PEI ^4^ [kcal/d]	PEI ^4^ [kcal/kg BW]	PEI-TEE [kcal]	TEE/PEI [Ratio]
Total (*n* = 169)	1179 ± 279	1447 ± 359	25 ± 6	1999 ± 400	36 ± 11	552 ± 496	0.76 ± 0.24
Sex							
Male (*n* = 45)	1381 ± 340	1697 ± 431	26 ± 7	1995 ± 433	32 ± 10	297 ± 525	0.89 ± 0.28
Female (*n* = 124)	1105 ± 212	1356 ± 280	25 ± 6	2000 ± 390	37 ± 11	644 ± 453	0.71 ± 0.20
*p* *	<0.001	<0.001	0.209	0.718	0.004	<0.001	<0.001
BMI ^5^ [kg/m^2^]							
Underweight (*n* = 78)	1121 ± 256 ^a^	1369 ± 33 ^a^	28 ± 6 ^a^	2073 ± 380	43 ± 9 ^a^	704 ± 457 ^a^	0.69 ± 0.21 ^a^
Normal (*n* = 51)	1124 ± 186 ^a^	1375 ± 246 ^a^	23 ± 4 ^b^	1908 ± 446	32 ± 8 ^b^	532 ± 500 ^a^	0.77 ± 0.25 ^a^
Overweight (*n* = 25)	1390 ± 321 ^b^	1731 ± 395 ^b^	23 ± 4 ^b^	1922 ± 347	26 ± 5 ^c^	191 ± 380 ^b^	0.91 ± 0.20 ^b^
Obesity (*n* = 15)	1315 ± 382 ^ab^	1620 ± 483 ^ab^	20 ± 5 ^b^	2049 ± 373	26 ± 6 ^bc^	429 ± 533 ^ab^	0.81 ± 0.25 ^ab^
*p* *	<0.001	<0.001	<0.001	0.070	<0.001	<0.001	<0.001
MUAC ^6^ [cm]							
Undernutrition (*n* = 43)	1043 ± 203	1258 ± 247	26 ± 5	1994 ± 426	42 ± 10	735 ± 489	0.67 ± 0.21
Normal (*n* = 126)	1225 ± 287	1511 ± 370	25 ± 6	2001 ± 393	33 ± 11	489 ± 485	0.79 ± 0.24
*p* *	0.026	0.044	0.002	0.203	<0.001	0.005	0.048
Level of activity							
In bed/sitting out (*n* = 110)	1107 ± 266 ^a^	1329 ± 319 ^a^	24 ± 5 ^a^	2005 ± 433	37 ± 11	676 ± 479 ^a^	0.70 ± 0.22 ^a^
Limited mobility (*n* = 35)	1300 ± 290 ^b^	1625 ± 362 ^b^	26 ± 6 ^b^	1991 ± 338	33 ± 10	366 ± 470 ^b^	0.84 ± 0.23 ^b^
Sedentary (*n* = 24)	1329 ± 201 ^b^	1728 ± 261 ^b^	28 ± 7 ^b^	1983 ± 338	33 ± 10	255 ± 407 ^b^	0.90 ± 0.22 ^b^
*p* *	<0.001	<0.001	0.003	0.782	0.090	<0.001	<0.001
Route of nutrition							
Oral (*n* = 143)	1202 ± 288	1477 ± 368	25 ± 6	2124 ± 261	38 ± 10	647 ± 465	0.71 ± 0.21
Enteral (*n* = 26)	1050 ± 181	1279 ± 248	24 ± 6	1310 ± 331	25 ± 8	31 ± 305	1.01 ± 0.24
*p* *	0.005	0.005	0.207	<0.001	<0.001	<0.001	<0.001
Served food consumption [%]							
<25 (*n* = 2)	1030 ± 336 ^a^	1299 ± 493 ^a^	25 ± 10	2130 ± 466 ^a^	40 ± 7 ^a^	831 ± 959 ^a^	0.65 ± 0.37 ^a^
25–50 (*n* = 36)	1071 ± 268 ^a^	1302 ± 336 ^a^	25 ± 6	2140 ± 236 ^a^	42 ± 10 ^a^	838 ± 422 ^a^	0.62 ± 0.17 ^a^
51–75 (*n* = 54)	1147 ± 212 ^a^	1405 ± 274 ^a^	26 ± 6	2178 ± 235 ^a^	40 ± 9 ^a^	773 ± 347 ^a^	0.65 ± 0.14 ^a^
>75 (*n* = 77)	1255 ± 307 ^ab^	1548 ± 395 ^ab^	24 ± 6	1804 ± 465 ^ab^	29 ± 9 ^ab^	256 ± 449 ^ab^	0.90 ± 0.24 ^ab^
*p* *	0.007	0.006	0.286	<0.001	<0.001	<0.001	<0.001

^1^ All variables were expressed as mean with standard deviation, while statistical tests were selected according to the type and distribution of the data; ^2^ REE—resting energy expenditure; ^3^ TEE—total energy expenditure; ^4^ PEI—planned energy intake; ^5^ Age-adopted BMI category cut-offs: underweight (BMI < 20 kg/m^2^ if <70 years and <22 kg/m^2^ if ≥70 years), normal body weight (BMI 20 to 25 kg/m^2^ if <70 years and 22 to 27 kg/m^2^ if ≥70 years), overweight (BMI 25 to 30 kg/m^2^ if <70 years and 27 to 30 kg/m^2^ if ≥70 years), obesity (BMI > 30 kg/m^2^); ^6^ MUAC—mid-upper arm circumference, undernutrition was defined as <23.5 cm for men and <22.0 cm for women. The values do not add to 100% because of rounding. * comparisons by *t*-test or Mann–Whitney U test (two groups), and by one-way ANOVA, Welch’s ANOVA, or Kruskal–Wallis test (≥3 groups); post hoc Tukey HSD, Bonferroni-adjusted *t*-tests, or Dunn’s test as appropriate. Different superscript letters (a, b, ab, c, bc) indicate statistically significant differences between groups based on post-hoc multiple comparison analysis (*p* < 0.05). Groups sharing at least one letter do not differ significantly.

**Table 3:** Distribution of residents (*n* = 106) across intake adequacy categories under hypothetical scenarios of served food consumption (percentage of prescribed energy intake, PEI), stratified by nutritional status (underweight vs. normal weight).

[%] of PEI		100	90	80	70	60	50
Underweight ^1^ (*n* = 66)							
<0.9 TEE	N	1	2	4	9	23	46
	%	1.5	3.0	6.1	13.6	34.8	69.7
0.9–1.1 TEE Adequate	N	2	5	12	23	24	12
	%	3.0	7.6	18.2	34.8	36.4	18.2
>1.1 TEE Adequate	N	63	59	50	34	19	8
	%	95.5	89.4	75.8	51.5	28.8	12.1
Normal weight ^1^ (*n* = 40)							
<0.9 TEE	N	1	2	5	12	21	31
	%	2.5	5.0	12.5	30.0	52.5	77.5
0.9–1.1 TEE Adequate	N	4	7	11	10	10	8
	%	10.0	17.5	27.5	25.0	25.0	20.0
>1.1 TEE	N	35	31	24	18	9	1
	%	87.5	77.5	60.0	45.0	22.5	2.5

^1^ Age-adopted BMI category cut-offs: underweight (BMI < 20 kg/m^2^ if <70 years and <22 kg/m^2^ if ≥70 years) and normal body weight (BMI 20–25 kg/m^2^ if <70 years and 22–27 kg/m^2^ if ≥70 years).

## Data Availability

The data presented in this study are available on request from the corresponding author due to privacy or ethical restrictions.

## Abbreviations

The following abbreviations are used in this manuscript:

BMI	Body mass index
IC	Indirect calorimetry
LTCFs	Long-term care facilities
MUAC	Mid-upper arm circumference
PAL	Physical activity level
PEI	Planned energy intake
REE	Resting energy expenditure
TEE	Total energy expenditure
